# Study on the Structure of Lignin Isolated from Wood Under Acidic Conditions

**DOI:** 10.3390/molecules30244705

**Published:** 2025-12-09

**Authors:** Andrzej Antczak, Aneta Skręta, Anna Kamińska-Dwórznicka, Klaudia Rząd, Arkadiusz Matwijczuk

**Affiliations:** 1 Institute of Wood Sciences and Furniture, Warsaw University of Life Sciences—SGGW, 159 Nowoursynowska St., 02-776 Warsaw, Poland; aneta_skreta@sggw.edu.pl; 2 Institute of Food Sciences, Warsaw University of Life Sciences—SGGW, 159C Nowoursynowska St., 02-776 Warsaw, Poland; anna_kaminska1@sggw.edu.pl; 3 Molecular Biophysics Institute, University of Life Sciences in Lublin, 13 Akademicka St., 20-950 Lublin, Poland; klaudia.rzad@up.lublin.pl (K.R.); arkadiusz.matwijczuk@up.lublin.pl (A.M.)

**Keywords:** lignin condensation, SEC, ATR-FTIR, SEM, acetylation, pine and poplar wood

## Abstract

Lignin obtained in acidic conditions is a waste product in various technological processes like sulfite pulping, organosolv pulping, or bioethanol production. Knowing the structure of the lignin enables its use in high-value-added applications. In this paper, the lignin structure isolated from *Pinus sylvestris* L. and *Populus deltoides* × *maximowiczii* wood in acidic conditions was investigated. Two methods of lignin isolation (Klason method and a method using a sulfuric and phosphoric acid mixture) were compared. Additionally, lignin acetylation was performed. The lignin samples were analyzed using different instrumental techniques, such as size exclusion chromatography (SEC), attenuated total reflection–Fourier transform infrared spectroscopy (ATR-FTIR), and scanning electron microscopy (SEM). Based on the studies carried out, it was found out that the lignin isolated from pine and poplar wood in acidic conditions had a highly condensed structure. This was evidenced by the high-weight average molar mass of lignin (up to 118,700 g/mol) and the precipitates, aggregates, and agglomerates on its surface. Moreover, the characteristic signals of condensed lignin in ATR-FTIR analysis (band with wavenumber of 767 cm^−1^) and their decrease/disappearance (band that usually occurs with a wavenumber of about 814 cm^−1^) were observed. Lignin acetylation and analysis in the 0.5% LiCl/DMAc system have proven particularly effective in the case of the condensed poplar lignin. The beneficial effect of lignin acetylation was confirmed by SEM analysis. The high-molecular-weight condensed lignin, despite some of its problematic properties connected mainly with solubility, is a valuable substance that can be used for different applications (carbon fibers or as an additive for thermoplastic blends), which was confirmed by the studies in this paper and the findings of other scientists.

## 1. Introduction

Lignin, as one of the most crucial organic compounds, is an integral component of both herbaceous and woody plants [1,2]. Lignin is responsible for structural functions within the plant, significantly impacting the mechanical strength of trees and facilitating water transport within the plant due to its hydrophobic characteristics [3,4]. Its presence in plants also acts as a barrier against pest and pathogen attacks [5,6].

As an aromatic compound, lignin possesses an amorphous three-dimensional structure composed of cross-linked chains of phenylpropane units [7]. Depending on its origin, lignin contains three distinct aromatic radicals: guaiacyl (G), syringyl (S), and p-hydroxyphenyl (H) [8,9].

Lignin derived from grasses is denoted as GSH, while lignin from softwood is designated as G and from hardwood as GS. The lignin content may vary depending on the wood species. According to Kumar et al. [10], softwood contains 25–35% lignin, whereas hardwood contains 18–25% of this component. Lignin often poses a barrier during the production of cellulose pulp [9] or biofuels [11]. It accounted for 95–98% of its intended use as an energy source during combustion [12]. Current trends classify lignin as a high-potential raw material due to its structure and properties, which predispose it to be utilized as a biopolymer, hybrid material, or feedstock to obtain valuable chemical substances [12,13].

**Figure 1:**
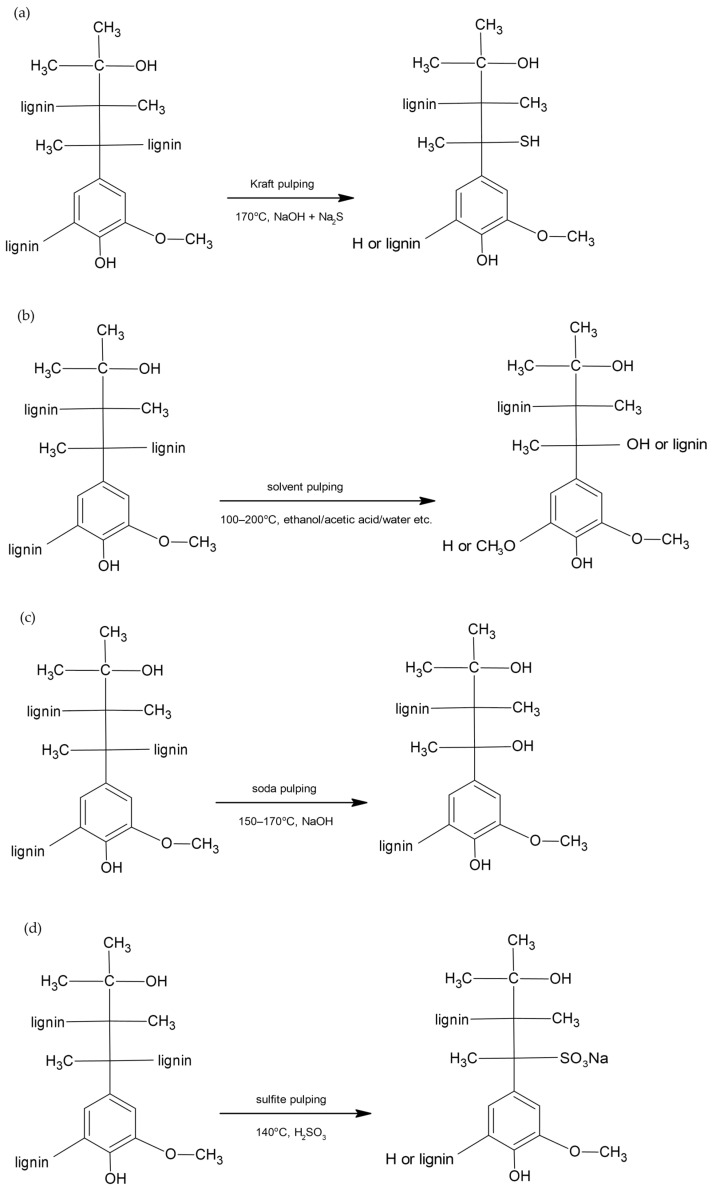
Lignin chemical reactions in commercial pulping processes. (**a**) Kraft pulping; (**b**) organosolv (solvent) pulping; (**c**) soda pulping; (**d**) lignosulfonate (sulfite) pulping.

Lignin, as a biopolymer with a complex structure, requires an appropriate isolation method. Many methods, using different chemicals, can be distinguished, which lead to different types of lignin, for example, Kraft lignin, organosolv lignin, soda lignin, lignosulfonate lignin, milled wood lignin (MWL), cellulolytic enzyme lignin (CEL), enzymatic mild acidolysis lignin (EMAL), or Klason lignin [14,15,16,17]. Of these, the first four are carried out on an industrial scale and have the greatest practical importance. The equations of chemical reactions occurring in the main pulping processes and leading to the production of different types of commercial lignin are shown in Figure 1 [18,19].

Each method has varying accuracy, which is connected with the purity and molar mass of the isolated lignin. However, the Klason lignin obtained by the TAPPI method [20] is considered highly reliable and is very often used for quantitative lignin analysis [21,22]. The limitations of this method are the condensation and repolymerization reactions of lignin in acidic conditions, which may hinder its solubility, the study of its structure, and its use to obtain valuable chemical substances [23,24]. The reason for this is the acid-catalyzed cleavage of α-O-4 ether bonds in the lignin structure, which generates benzylic carbocations. These highly reactive carbocations then undergo electrophilic substitution with electron-rich aromatic rings in other lignin units, forming new carbon–carbon bonds that lead to repolymerization and condensation [25,26]. Factors that accelerate lignin condensation and repolymerization reactions include elevated temperatures, acid concentrations, and reaction times. The structure of lignin also matters. Pine lignin is composed primarily of guaiacyl units, which react slowly in condensation reactions, but the resulting product is stable, durable, and has a high molar mass. Poplar lignin, on the other hand, is composed mainly of syringyl units (with a lower share of guaiacyl units), which react very quickly in such reactions, and the resulting condensation products are unstable and easily degraded, forming products with a lower molar mass [27].

From the point of view of lignin applications, this biopolymer has numerous interesting directions. Particularly important are the environmentally friendly applications that serve as sustainable alternatives to fossil-based materials across various industries. Utilizing lignin, often a byproduct of the pulp and paper industry that was traditionally burned for energy, reduces waste and greenhouse gas emissions. Key applications include the production of bioplastics, formaldehyde-free and non-toxic bioadhesives, carbon fibers, controlled-release fertilizers, water filters, bioasphalts, biofuels, biochemicals, and as an antioxidant in cosmetics and pharmaceuticals [12,28,29,30]. By valorizing lignin, industries can transition away from a dependence on fossil fuels and move toward a more sustainable, low-carbon circular bioeconomy.

In order to determine the suitability of lignin for high-value-added applications, it is necessary to analyze its molar mass and examine its structure to assess its reactivity and physicochemical properties [31,32,33]. Various methods are used to analyze lignin structure [21,34,35,36,37]. Among them, the following can be distinguished: size exclusion chromatography (SEC), Fourier transform infrared spectroscopy (FTIR), and scanning electron microscopy (SEM). FTIR is a technique that can be utilized for detecting the functional groups of lignin and its impurities in the form of polysaccharides. In turn, SEM is an effective technique that can be used to visualize lignin structures in high resolution, providing information about the surface morphology. Moreover, FTIR and SEM can be used to analyze the phenomenon of lignin condensation in various acidic isolation processes, which can be crucial information for its practical use, especially in industrial conditions [38,39]. SEC analysis is the most commonly used technique for the separation and then qualitative and quantitative determination of natural and synthetic polymers. This method enables the obtaining of the molar mass distribution (MMD) of polymers, the average molar masses, such as the weight average molar mass (M_w_) or the number average molar mass (M_n_), and the polydispersity index (PDI). Most often, the molar masses of lignin isolated from plant raw materials range from 1000 to 20,000 g/mol [40]. However, in specific conditions (especially due to the condensation and repolymerization reactions of lignin), this parameter is much higher and can even be up to about 100,000 g/mol [16,24].

The earlier-mentioned lignin applicability is limited by its heterogeneity, variability, and low reactivity [41]. These problems may be significantly overcome if the structure of lignin is modified during the derivatization process. One of the most commonly used methods of lignin derivatization is acetylation. Acetylation improves the solubility of the lignin in organic solvents and also can be used as a pretreatment step when a soluble form of a lignin is required in manufacturing processes [42]. Moreover, in the case of the SEC analysis of acetylated lignin, the occurrence of the adsorption phenomenon can be significantly reduced [33].

The aim of the research in this paper was to investigate the structure of the lignin isolated from pine and poplar wood in acidic conditions. Acid methods, such as the model Klason method, are very often used to isolate a lignin and determine its quantity. Moreover, lignins in acidic conditions can be obtained as a waste product during industrial technological processes, such as sulfite pulping or bioethanol production. Also important and interesting is the comparison of the structure of the lignin extracted from *Pinus sylvestris* L. and *Populus deltoides* × *maximowiczii* wood by instrumental techniques (SEC, FTIR, or SEM). These species can be used in industrial technological processes due to their available raw material base. *Pinus sylvestris* L. is a well-known conifer with a wide natural range in Europe (including Poland) and Asia. *Populus deltoides* × *maximowiczii*, on the other hand, is a deciduous hybrid species of fast-growing poplar, purpose-bred in Europe (including Poland). Its advantages include its ability to adapt to unfavorable habitat conditions, suitability for plantation cultivation, and use for soil phytoremediation. Unfortunately, the available literature lacks publications which focus on the analysis of the structure (by SEC, FTIR, and SEM techniques) of the lignin isolated from these materials in acidic conditions. The novelty of this paper is indicated by the SEC, ATR-FTIR, and SEM techniques, which show that the lignin isolated from *Populus deltoides* × *maximowiczii* and *Pinus sylvestris* L. wood in acidic conditions has a highly condensed structure. The condensed lignin is a valuable substance that can be used for different applications, like carbon fibers or as an additive for thermoplastic blends. Hence, taking into account the possibility of the occurrence of a raw material base of a lignin obtained by acid methods, it seems to be important from a scientific and practical point of view to analyze its structure. Knowing the structure of a lignin will enable its use in high-value-added applications.

## 2. Results and Discussion

### 2.1. SEC Analysis

The SEC analysis was conducted on lignin samples, which were isolated from poplar and pine wood using acid-based methods, and the samples underwent derivatization through acetylation. The results (M_n_, M_w_, and PDI) of non-acetylated and acetylated lignin are compared and presented in Table 1. The solvent used was 0.5% LiCl/DMAc.

**Figure 2:**
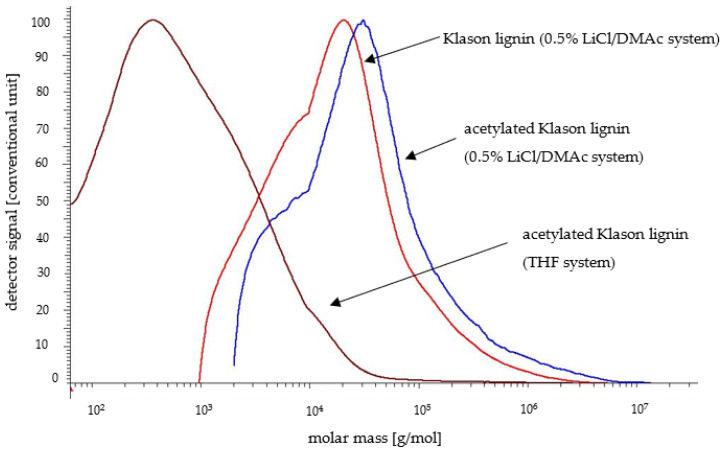
Molar mass distributions of non-acetylated and acetylated Klason lignin isolated from poplar wood determined in 0.5% LiCl/DMAc and THF systems.

**Figure 3:**
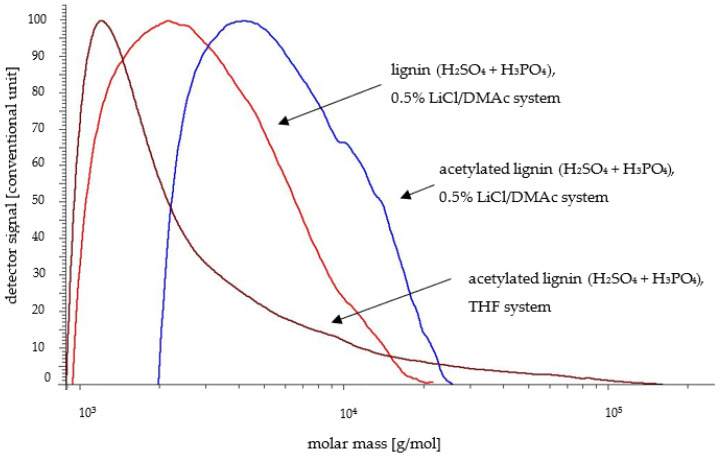
Molar mass distributions of non-acetylated and acetylated lignin (H_2_SO_4_ + H_3_PO_4_) isolated from pine wood determined in 0.5% LiCl/DMAc and THF systems.

The results concerning the non-acetylated lignins from both wood species were discussed in an earlier article [24]. In the present publication, the SEC parameters of lignin samples after the acetylation process determined in a 0.5%LiCl/DMAc system are shown and compared in Table 1. The studies performed on both the poplar Klason lignin and the lignin isolated using a sulfuric acid and phosphoric acid mixture led to higher M_n_ and M_w_ values compared to non-acetylated samples. The most significant increase was observed for the Klason lignin, where M_w_ rose from 53,577 g/mol to 118,700 g/mol in the 0.5% LiCl/DMAc system. In the case of the lignin isolated with the acid mixture, the increase of M_n_ and M_w_ after derivatization was high but not so significant as for the Klason lignin. The results were similar for the lignin isolated from pine wood and subjected to acetylation (Table 1). In this case, the M_n_, M_w_, and PDI values were also higher when using the Klason method compared to the acid mixture method. However, the derivatization of pine Klason lignin did not result in an increase comparable to that observed in the poplar lignin. A slight increase was noted for M_w_ and PDI values. In contrast, for the lignin obtained with the sulfuric and phosphoric acids mixture, M_n_ increased from 2278 g/mol to 4595 g/mol, and M_w_ increased from 3513 g/mol to 6327 g/mol. These observations were probably due to the condensation and repolymerization reactions of lignins in acidic conditions and also the increased solubility of this component after acetylation, which was confirmed by the molar mass distributions presented in Figure 2 and Figure 3. Acetylated lignin was more soluble in the 0.5% LiCl/DMAc system, especially high-molecular-weight poplar lignin fractions, which could be taken into account in this case. The reason for this can be a change in the polarity of the lignin molecule and the disruption of hydrogen bonds. Lignins with acetyl groups are less polar, which effectively block the formation of strong intra- and intermolecular hydrogen bonds. The absence or limited occurrence of such interactions in acetylated lignin facilitates the penetration of solvent molecules into the lignin structure, increasing its solubility. However, in the case of pine lignin, which is even more condensed than poplar lignin, no such large increase in the molar masses was observed. Furthermore, the molar mass values obtained (especially M_w_) for pine lignin were lower than those obtained for poplar lignin. This is likely due to the limited solubility of highly condensed pine lignin in a 0.5% LiCl/DMAc system. High-molecular-weight pine lignin fractions are especially less soluble, hence the obtained M_w_ values were lower than for poplar lignin. In this case, it is worth noting that the pine lignin generally has a more compact structure than the poplar lignin, because of the higher condensation and cross-linking degree. The greater number of C-C bonds in pine lignin makes its macromolecules less flexible and more difficult to interact with solvent molecules. Furthermore, this kind of pine lignin structure may limit the availability of -OH groups for acetylation, and the post-acetylated structure remains less susceptible to solvent penetration than the more “open” structure of the poplar lignin.

The literature data rarely mention studies that allow for a direct comparison of the molecular weight values of acetylated lignins soluble in a 0.5% LiCl/DMAc system. Additionally, the use of this solvent is not commonly reported in the context of lignin dissolution, as it is typically employed for samples that have not undergone derivatization. Moreover, the literature reports very often refer to lignin isolated by other methods and hence characterized by different parameters. Ringena et al. [43] obtained different results for non-acetylated samples analyzed in a LiCl/DMAc system and reported that the M_w_ of steam explosion aspen lignin was 35,000 g/mol, the M_n_ was 2200 g/mol, and the PDI was 15.9. Esakkimuthu [44] studied five different lignin samples in the SEC-LiCl/DMAc system. The obtained M_w_ range for underivatized lignin was from 4080 to 25,270 g/mol, while, for acetylated lignin, it was from 5690 to 79,600 g/mol. It was found, analogously to this work, that acetylated lignin had higher M_w_ values than underivatized lignin.

An SEC analysis was also performed using THF as a solvent. According to the literature’s knowledge, this approach is specifically dedicated to acetylated samples. The corresponding values are presented in Table 2.

The M_n_, M_w_, and PDI values presented in Table 2 for acetylated poplar lignin samples analyzed using THF as a solvent were significantly higher for Klason lignin than for the method using the acid mixture. In the case of pine lignin, regardless of the method used, the values were more similar. In terms of the solvent choice for the SEC analysis, the molar mass values obtained using 0.5% LiCl/DMAc were also higher than for THF. These findings were also visible for the molar mass distributions of acetylated lignin isolated from poplar and pine wood analyzed in the THF system (Figure 2 and Figure 3). The molar mass distributions of lignin isolated by the Klason method and using the acid mixture determined in THF were significantly shifted towards lower molar mass fractions. This is confirmed by the results of Gosselink et al. [45], who studied the molar mass distribution of organosolv lignin in a similar solvent system (DMF/LiCl 0.2 M and THF). According to these findings, the weight average molar mass was lower in THF than in DMF/LiCl.

The values reported by Sannigrahi et al. [46] for the acetylated milled wood lignin obtained from *Pinus taeda* L. determined in a THF system were 13,500 g/mol (M_w_), 7590 g/mol (M_n_), and 1.8 (PDI). Compared to the acetylated pine lignin samples in this paper (Table 2), the literature molar mass values were higher. In turn, Baumberger et al. [33] studied acetylated softwood Kraft lignin and obtained results similar to those presented in this work. Based on the SEC analysis performed in the THF system, they found that the lignin had the following parameters: M_w_ = 5621 g/mol, M_n_ = 1285 g/mol, and PDI = 4.4. Also, the findings of Kim et al. [47] for acetylated milled wood lignin obtained from *Populus alba* × *Populus glandulosa* analyzed in THF, especially for M_w_ (10,002 g/mol), were very similar, although M_n_ (4060 g/mol) and PDI (2.5) were different.

According to the authors’ opinion, despite the quite good agreement with the literature data, the results obtained by the SEC method in the THF system for condensed poplar and pine lignin were underestimated. In this case, the method in the 0.5% LiCl/DMAc system proved to be better, especially after acetylation and for poplar lignin obtained under acidic conditions. However, regarding pine lignin, due to its higher degree of condensation and very high molecular weight, the problem of its SEC analysis remains unresolved.

To sum up, based on the SEC studies, the determined molar masses and polydispersity index of acetylated lignin in the 0.5% LiCl/DMAc system were the most reliable, as evidenced by the obtained molar mass distributions shifted towards fractions with higher molar masses. This method has proven particularly effective in the case of the condensed lignin obtained from poplar wood under acidic conditions.

### 2.2. ATR-FTIR Analysis

**Figure 4:**
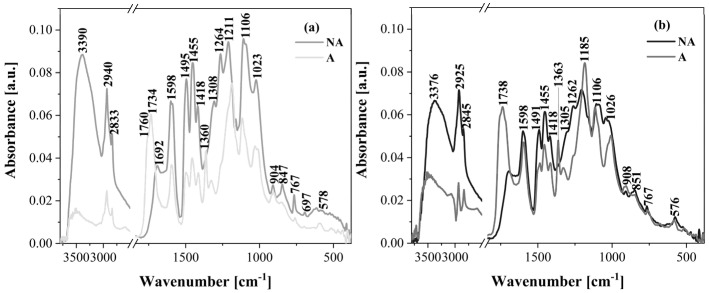
ATR-FTIR spectra of non-acetylated (NA) and acetylated (A) lignin isolated from poplar wood by acid methods. (**a**) Klason lignin; (**b**) lignin (H_2_SO_4_ + H_3_PO_4_).

**Figure 5:**
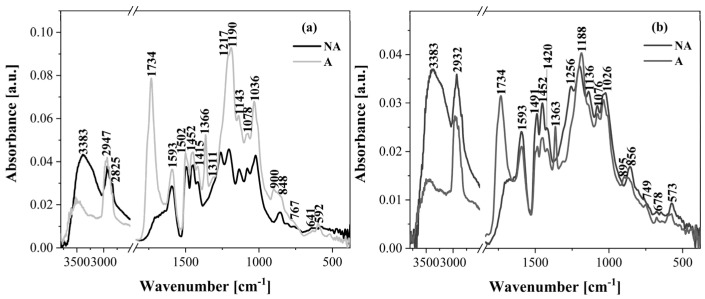
ATR-FTIR spectra of non-acetylated (NA) and acetylated (A) lignin isolated from pine wood by acid methods. (**a**) Klason lignin; (**b**) lignin (H_2_SO_4_ + H_3_PO_4_).

The ATR-FTIR technique was used to characterize the structure of the lignin isolated from poplar and pine wood using two different acid methods. Additionally, the obtained ATR-FTIR spectra (Figure 4 and Figure 5) of the non-acetylated lignin were compared with those of the acetylated lignin.

The band assignments were made based on the literature findings [48,49,50] and are presented in Table 3. The main differences in the results obtained from ATR-FTIR analysis included signals resulting from the acetylation reaction of the lignin. As a result of the acetylation of the lignin isolated from poplar and pine wood, a decrease in the intensity of the band originating from the stretching vibration of the OH group (3390 cm^−1^, 3376 cm^−1^, and 3383 cm^−1^) and the appearance of a strong band corresponding to the carbonyl group (C=O unconjugated) at wavenumbers of 1734 cm^−1^ and 1738 cm^−1^ were observed (Figure 4 and Figure 5). Moreover, the spectrum of the acetylated lignin showed the presence of additional characteristic bands from the acetyl group, i.e., C=O stretching vibrations of high intensity (1185 cm^−1^, 1190 cm^−1^, and 1188 cm^−1^) and a C-H bond vibration of lower intensity (1360 cm^−1^, 1363 cm^−1^, and 1366 cm^−1^) (Figure 4 and Figure 5; Table 3). In contrast, this spectrum lacked the visible bands at 1264 cm^−1^, 1262 cm^−1^, and 1256 cm^−1^ assigned to the C-O stretching vibration (methoxyl group of guaiacyl unit) and the bands at 1211 cm^−1^ and 1217 cm^−1^ associated with the C-O stretching vibration (phenolic hydroxyl group), which were strong signals in the spectrum of non-acetylated lignin. These spectral changes confirm the successful acetylation of lignin.

The remaining signals described in Table 3 did not differ significantly and occurred in each lignin analyzed, regardless of the acid method used. Hence, the presence of methoxy groups (bands 2947–2925 cm^−1^ and 1036–1023 cm^−1^), phenolic hydroxyl groups (1211 cm^−1^ and 1217 cm^−1^), and aromatic rings (1598 cm^−1^ and 1593 cm^−1^, 1502–1491 cm^−1^, 1420–1415 cm^−1^, and 908–895 cm^−1^) were identified. These features were observed in lignin samples from both poplar and pine. Such lignin can be a source of valuable organic substances [16,28,41]. Examples of such substances include methanol, benzene, toluene, cyclohexane, phenol, vanillin, or styrene.

In most cases, the obtained ATR-FTIR spectra contained the same signals, differing only in their intensity (Figure 4 and Figure 5). Significant differences were observed in the band at the wavenumber of 1106 cm^−1^ of the syringyl unit (C-H deformation vibration) occurring only in the poplar lignin (Figure 4; Table 3) and the band at 1143 cm^−1^ or 1136 cm^−1^ of the guaiacyl unit (C-H deformation vibration) occurring only in the pine lignin (Figure 5; Table 3). Also, the bands at 1256 cm^−1^, 1264 cm^−1^, and 1262 cm^−1^ assigned to the C-O stretching vibration in the guaiacyl unit observed in non-acetylated poplar and pine lignin and the bands at 847 cm^−1^, 851 cm^−1^, 848 cm^−1^, and 856 cm^−1^ from the C-H out of plane at positions 2, 5, and 6 of the guaiacyl unit are particularly characteristic, as they support the observations of other scientists that poplar lignin has a mixed syringyl–guaiacyl structure (with a predominance of syringyl units), while pine is almost exclusively composed of guaiacyl fragments [16,51].

Another interesting aspect that can be monitored by FTIR is the phenomenon of lignin condensation, which can occur during its isolation in acidic conditions. In the case of the lignin samples analyzed in this work, the signal from condensed lignin was also identified. This is a band at a wavenumber of 767 cm^−1^ (Figure 4), visible for both the non-acetylated and acetylated lignin isolated from poplar wood by acid methods. According to the data reported by Fiskari et al. [38], this band corresponds to a shifted signal of the C-H out of plane deformation vibration in the syringyl unit of the condensed lignin. Such a condensed lignin, isolated from hardwoods, has a characteristic absorption band around 780 cm^−1^. In the case of lignins from softwood species, the presence of lignin condensation is indicated by a significant reduction or disappearance of the band at approximately 814 cm^−1^, corresponding to the C-H out of plane deformation vibration in the guaiacyl unit [39]. The ATR-FTIR spectra presented in Figure 5 confirmed this hypothesis, as no signals around 814 cm^−1^ were visible. Hence, the results obtained from the ATR-FTIR analysis suggest that the lignin isolated from poplar and pine wood by acid methods exhibits the properties of a condensed lignin. This was also confirmed by earlier research carried out on the same lignin samples [24]. As a result of ball milling, the progressive processes of degradation, repolymerization, and condensation of lignin were observed.

### 2.3. SEM Analysis

**Figure 6:**
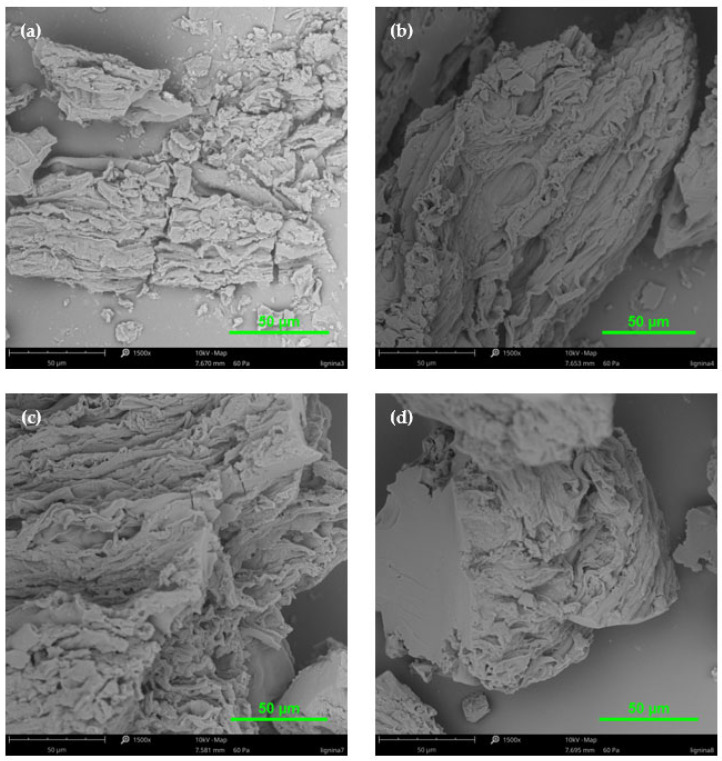
SEM images of lignin isolated from poplar wood by acid methods: (**a**) non-acetylated Klason lignin; (**b**) non-acetylated lignin (H_2_SO_4_ + H_3_PO_4_); (**c**) acetylated Klason lignin; (**d**) acetylated lignin (H_2_SO_4_ + H_3_PO_4_).

**Figure 7:**
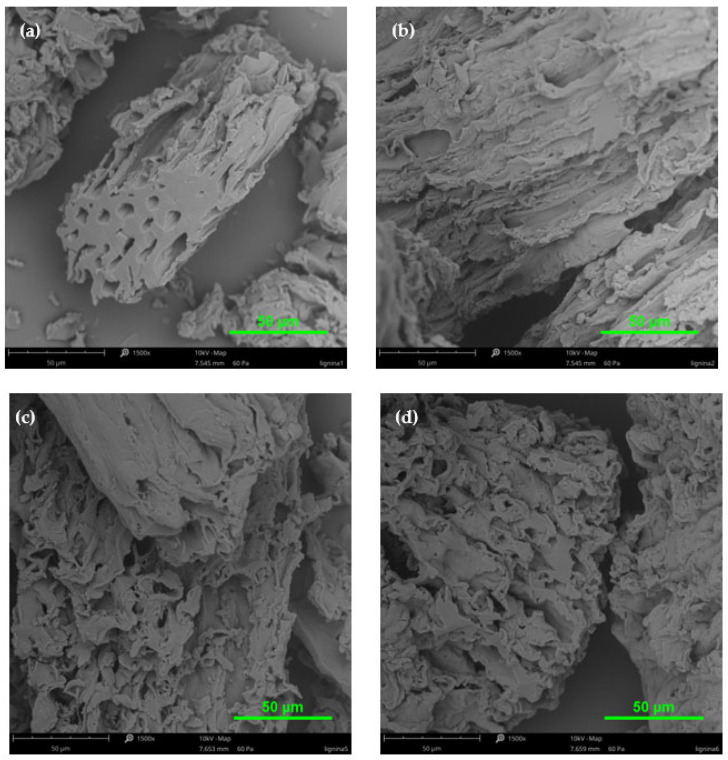
SEM images of lignin isolated from pine wood by acid methods: (**a**) non-acetylated Klason lignin; (**b**) non-acetylated lignin (H_2_SO_4_ + H_3_PO_4_); (**c**) acetylated Klason lignin; (**d**) acetylated lignin (H_2_SO_4_ + H_3_PO_4_).

The SEM technique was used to visualize lignin particles’ structures in high resolution and provide information about their surface morphology. This technique was used to complement the analytical methods employed in this work. Using SEM, it was possible to directly observe the surface morphology and microstructure of lignins. In the studies, lignin samples isolated from poplar and pine wood in acidic conditions were compared. Additionally, the influence of acetylation processes on the lignin surface structure was studied. The images from the SEM analysis are presented in Figure 6 and Figure 7.

Based on the SEM images (Figure 6 and Figure 7), it can be observed that lignin isolated by the Klason method was more degraded and less condensed than by the method using a mixture of sulfuric and orthophosphoric acids. For both types of lignins (especially without acetylation and for the poplar lignin), we observed crushed and small particles. On the other hand, for the pine lignin, the particles were bigger and less degraded. Moreover, the surfaces of all the tested lignin samples were characterized by numerous pores, folds, and grooves.

According to Fiskari et al. [38,39], precipitates on the surface of cellulose fiber came from condensed lignin. Analogous observations were confirmed by Pua et al. [52] during the SEM analysis of Kraft lignin samples. After acetylation, both types of lignins looked more similar to the bigger part of aggregates and even agglomerates, which was already observed in earlier research [53,54].

The surface images of the analyzed lignin samples (isolated from both poplar and pine wood) from different acid methods became more uniform and homogeneous as a result of acetylation. The SEM analysis confirmed that the tested lignin samples, isolated in acidic conditions, showed the characteristics of condensed lignins. In particular, on the one hand, the occurrence of precipitates on the lignin surface confirmed this statement, and also the presence of aggregates or agglomerates.

The high-molecular-weight condensed lignin is also a valuable substance that can be used for different applications. Such a lignin is a good source of polyols for thermoplastic polyester and polyurethane synthesis. A lignin with a high molecular weight provides better mechanical parameters for materials and exhibits a higher loading capacity as a low-cost extender for rigid polyurethane foams [16]. The use of lignins may also be important for the production of carbon fibers. Low-molecular-weight lignins increase the brittleness of carbon fibers and cause difficulties in spinning them [55]. Moreover, recent studies have shown that condensed lignins are an important additive for thermoplastic blends, copolymers, and adhesives [56,57,58].

The condensed lignin, despite the existence of many interesting directions for its use, encounters a number of significant limitations, mainly resulting from its complex and diverse chemical structure. The main barriers include difficulties in obtaining a homogeneous raw material with the constant parameters necessary for industrial applications, low chemical reactivity, problems with solubility and processing, and the occurrence of technological limitations. Despite advances in research (e.g., use in solar cells and polyols), many applications remain in the laboratory or pilot phase, and commercialization requires further research and development. Intensive research is currently underway on the chemical modifications of lignins to overcome these limitations and enable their wider, more valuable use. From a scientific and practical point of view, it is important to know the structure of a lignin. By knowing its exact structure, one can determine the directions of its applications, which may contribute to the development of technology on an industrial scale in the future.

## 3. Materials and Methods

### 3.1. Material Characteristics

The study used wood samples from a species representing hardwood—Maksymowicz poplar (*Populus deltoides* × *maximowiczii*)—and softwood—Scots pine (*Pinus sylvestris* L.). The 7-year-old poplar wood was obtained from the experimental field in Wolica, which belongs to the Department of Plant Genetics, Breeding, and Biotechnology at the Institute of Biology, SGGW, in Warsaw. The 30-year-old pine wood (a mixture of the sapwood and heartwood zones) came from the Białowieża Forest District located in the Białowieża Forest in the Podlaskie Voivodeship. The collected material was obtained from debarked logs and reduced to a fraction size of 0.43–1.02 mm.

The chemical composition of the material was analyzed in earlier studies and described by Skręta and Antczak [24]. The *Populus deltoides* × *maximowiczii* wood consisted of 2.1% extractives, 20.5% total lignin (including 2.1% of acid-soluble lignin), 50.4% cellulose, 32.7% hemicelluloses, and 0.6% mineral substances, whereas the *Pinus sylvestris* L. wood consisted of 2.0% extractives, 29.0% total lignin (including 0.4% of acid-soluble lignin), 56.5% cellulose, 19.0% hemicelluloses, and 0.2% mineral substances. The presented chemical composition results refer to the dried and extracted material. All of the chemical substances were of analytical grade and purchased from Sigma-Aldrich (Taufkirchen, Germany).

### 3.2. Research Methods

**Figure 8:**
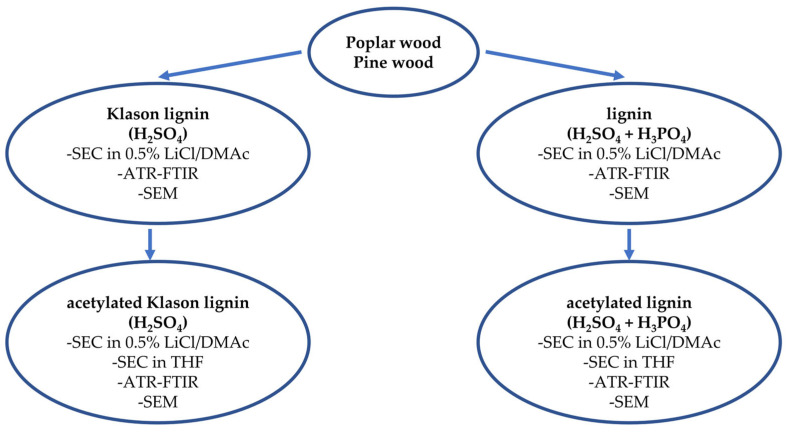
General scheme of research.

The general scheme of research was illustrated in Figure 8. The following subsections describe in detail the course of the individual stages presented in the diagram.

#### 3.2.1. Lignin Isolation Method in Acidic Conditions

The lignin was isolated from poplar and pine wood species, which were previously dried and subjected to an extraction process. To remove extractives, a mixture of chloroform and ethanol in a weight ratio of 93:7 was used [59,60]. The procedure was conducted for 10 h. Acidic lignin isolation methods were employed according to the Klason method using 72 wt.% sulfuric acid (VI) (H_2_SO_4_) [20] and the method which involved the use of a mixture of 75 wt.% H_2_SO_4_ and 89 wt.% orthophosphoric acid (H_3_PO_4_) [61]. After isolation, the lignin samples were washed with distilled water to achieve a pH of 6 and then dried to a constant mass at 103 °C ± 2 °C.

#### 3.2.2. Lignin Acetylation

The derivatization was carried out on both types of lignin samples according to the method proposed by Esakkimuthu et al. [62], with some modifications. A sample of 100 mg of dried lignin weighed in a glass screw-cap tube with a volume of 10 cm^3^ was acetylated with a mixture of 2.5 cm^3^ of pyridine and 2.5 cm^3^ of acetic anhydride at room temperature for 24 h with using a rotary mixer (RM-2M, Elmi, Calabasas, CA, USA). The acetylation process was stopped by adding 40 cm^3^ of 50 wt.% aqueous methanol solution, followed by filtration through the G3 Schott funnel. After methanol solution separation, the acetylated lignin was washed three times with toluene (each portion of 40 cm^3^) and once with methanol (40 cm^3^). Finally, the acetylated lignin was oven dried at 60 °C for 24 h.

#### 3.2.3. Acetylated Lignin Dissolution for SEC Analysis

The acetylated lignin samples were dissolved in tetrahydrofuran (THF) for SEC analysis to determine the lignin MMD and other important parameters that describe polymer properties, like M_w_, M_n_, and PDI. For comparison, the acetylated lignin samples were also dissolved in 0.5 wt.% LiCl/N,N-dimethylacetamide (DMAc) and analyzed in the SEC system.

The dissolution procedure in THF was as follows:

- A total of 10 mg of acetylated lignin sample was weighed in an Eppendorf tube;
- Next, 1 cm^3^ of THF was added, and the test tubes were closed;
- The lignin sample dissolution over 1 h was carried out using a rotary mixer (RM-2M, Elmi, Calabasas, CA, USA);
- After dissolution, the samples were filtered using a 0.22 µm nylon syringe filter;
- Finally, for each sample, three SEC analyses were performed.

The dissolution procedure in 0.5% LiCl/DMAc was as follows:

- A total of 10 mg of acetylated lignin sample was weighed in a glass screw-cap tube with a volume of 10 cm^3^;
- Next, 5 cm^3^ of 0.5% LiCl/DMAc was added, and the test tubes were screwed tight;
- The lignin sample dissolution over 7 days was carried out using a rotary mixer (RM-2M, Elmi, Calabasas, CA, USA);
- After dissolution, the samples were filtered using a 0.22 µm nylon syringe filter;
- Finally, for each sample, three SEC analyses were performed.

#### 3.2.4. SEC Analysis

The SEC analysis was conducted using a high-performance liquid chromatography (HPLC) system, specifically the LC-20AD model produced by Shimadzu, which was connected to a differential refractive index detector (RID 10A, Shimadzu, Kyoto, Japan). The conditions for SEC analysis were described in detail by Skręta and Antczak [24]. For the lignin samples dissolved in THF and 0.5% LiCl/DMAc, the following chromatographic separation conditions were applied: eluent in accordance with the solvent used; two PLgel MIXED-B, 10 µm, 7.5 × 300 mm columns (Agilent, Palo Alto, CA, USA) connected with PLgel guard column; oven temperature: 35 °C (for analysis in THF) and 80 °C (for analysis in 0.5% LiCl/DMAc); flow rate of the eluent: 1 cm^3^/min; injection volume of sample: 0.2 cm^3^. Eight narrow dispersion polystyrene standards (Agilent, Palo Alto, CA, USA) were used for conventional column calibration. The molar mass ranges of the polystyrene standards were as follows: 9570–6,570,000 g/mol.

#### 3.2.5. ATR-FTIR Analysis

An IRSpirit spectrophotometer (Shimadzu, Tokyo, Japan) was employed to perform attenuated total reflection–Fourier transform infrared spectroscopy (ATR-FTIR) measurements on acetylated and non-acetylated lignin samples originating from poplar and pine wood. A Zn-Se crystal with a 45° geometry was used as the ATR attachment, enhancing the internal reflections of the laser beam. The lignin samples were placed on the crystal. The attachment significantly improved the measurement accuracy by allowing for a precise control of the contact between the crystal and the sample, while also facilitating pressure adjustments. Each measurement involved 24 scans per sample, with the software automatically averaging the resulting spectra. The crystal was thoroughly cleaned with ultrapure solvents before and after each measurement, which were sourced from Sigma-Aldrich (Poznań, Poland). The scans were performed over a spectral range of 450 to 3600 cm^−1^, with a resolution of 2 cm^−1^. Additionally, each spectrum was averaged with five previous measurements to mitigate issues related to sample homogeneity. The measurements were carried out at room temperature. All ATR-FTIR analyses were conducted at the Molecular Biophysics Institute of the University of Life Sciences in Lublin, and the spectra were processed using Grams AI software (version 9.1) from ThermoGalactic Industries (San Jose, CA, USA).

#### 3.2.6. SEM Analysis

Acetylated and non-acetylated lignin samples originating from poplar and pine wood were also analyzed by SEM. Analyzed samples were first covered with a layer of gold using a 108 Auto Sputter Coater (Cressington, Watford, UK), according to the methodology used before for this type of a material [63]. Particle morphology was described based on images taken with the Phenom XL scanning electron microscope (Thermo Fisher Scientific, Waltham, MA, USA) at a magnification 1500× at an accelerating voltage of 10 kV.

## 4. Conclusions

Based on performed studies, the following conclusions were drawn:

1. Lignin isolated from poplar and pine wood in acidic conditions had a highly condensed structure, which was confirmed by SEC, ATR-FTIR, and SEM techniques.
2. The SEC analysis of acetylated lignin in the 0.5% LiCl/DMAc system indicated that the determined parameters (M_n_, M_w_, and PDI) were more reliable than in THF regardless of the isolation method. The weight of the average molar mass was up to 118,700 g/mol and was much higher for the acetylated lignin isolated in acidic conditions from poplar wood than from pine wood. This is likely due to the limited solubility of highly condensed pine lignin in the 0.5% LiCl/DMAc system. Particularly interesting was the fact that, in this system after acetylation, more reliable results were obtained for condensed poplar lignin.
3. The ATR-FTIR analysis confirmed that the lignin acetylation reaction was successful, and in all lignin spectra characteristic signals corresponding to methoxy groups, phenolic hydroxyl groups, and aromatic rings were observed. Moreover, this technique is a useful method for monitoring lignin condensation phenomena. Lignin obtained in acidic conditions also can be used for different value-added applications.
4. The SEM technique confirmed that the tested lignin samples, isolated in acidic conditions, showed the characteristics of condensed lignin. It was especially visible for pine lignin, as its particles were bigger and less degraded. The occurrence of precipitates on the lignin surface and also the presence of aggregates and agglomerates proved these observations. After acetylation, the surface image of lignin became more uniform and homogeneous.
5. Condensed lignin, despite some of its limitations connected mainly with its complex and diverse chemical structure, is a valuable substance that can be used for different applications (carbon fibers or as an additive for thermoplastic blends). However, to be able to use lignin on an industrial scale in the future, further research and development are needed.

## Figures and Tables

**Table 1:** The SEC parameters determined in 0.5%LiCl/DMAc system of non-acetylated and acetylated lignin isolated from poplar and pine wood by acid methods.

Lignin	M_n_ [g/mol]	M_w_ [g/mol]	PDI
**Poplar wood**			
Klason lignin (H_2_SO_4_)	8339 ± 233 *	53,577 ± 2251 *	6.4 ± 0.3 *
Lignin (H_2_SO_4_ + H_3_PO_4_)	4126 ± 443 *	8530 ± 618 *	2.1 ± 0.1 *
Acetylated Klason lignin (H_2_SO_4_)	11,470 ± 1604	118,700 ± 4597	10.5 ± 1.2
Acetylated lignin (H_2_SO_4_ + H_3_PO_4_)	5153 ± 586	10,897 ± 617	2.1 ± 0.1
**Pine wood**			
Klason lignin (H_2_SO_4_)	4653 ± 824 *	6092 ± 358 *	1.3 ± 0.3 *
Lignin (H_2_SO_4_ + H_3_PO_4_)	2278 ± 337 *	3513 ± 425 *	1.5 ± 0.0 *
Acetylated Klason lignin (H_2_SO_4_)	4478 ± 625	7492 ± 214	1.7 ± 0.3
Acetylated lignin (H_2_SO_4_ + H_3_PO_4_)	4595 ± 488	6327 ± 465	1.4 ± 0.1

* Skręta and Antczak [24].

**Table 2:** The SEC parameters determined in THF system of acetylated lignin isolated from poplar and pine wood by acid methods.

Lignin	M_n_ [g/mol]	M_w_ [g/mol]	PDI
**Poplar wood**			
Acetylated Klason lignin (H_2_SO_4_)	327 ± 19	10,846 ± 546	33.2 ± 0.4
Acetylated lignin (H_2_SO_4_ + H_3_PO_4_)	170 ± 26	1783 ± 161	10.6 ± 1.7
**Pine wood**			
Acetylated Klason lignin (H_2_SO_4_)	1885 ± 73	6193 ± 32	3.3 ± 0.1
Acetylated lignin (H_2_SO_4_ + H_3_PO_4_)	1923 ± 30	5251 ± 153	2.7 ± 0.1

**Table 3:** Band assignments from ATR-FTIR analysis of non-acetylated and acetylated lignin isolated from poplar and pine wood by acid methods.

Lignin Source	Peak Wavenumber [cm^−1^]				Band Assignment
	Klason Lignin (H_2_SO_4_)	Acetylated Klason Lignin (H_2_SO_4_)	Lignin (H_2_SO_4_ + H_3_PO_4_)	Acetylated Lignin (H_2_SO_4_ + H_3_PO_4_)	
Poplar wood	3390	3390 ↓ ^1^	3376	3376 ↓	O-H stretching
	2940	2940	2925	2925	C-H stretching (methoxyl group)
	2833	2833	2845	2845	C-H stretching (methyl and methylene groups)
	-	1734 ↑ ^2^	-	1738 ↑	C=O unconjugated (carbonyl group)
	1598	1598	1598	1598	Aromatic skeletal vibrations
	1495	1495	1491	1491	Aromatic skeletal vibrations
	1455	1455	1455	1455	C-H deformation (methyl and methylene groups)
	1418	1418	1418	1418	Aromatic skeletal vibrations
	-	1360 ↑	-	1363 ↑	C-H bond (acetyl group)
	1264	-	1262	-	C-O stretching (guaiacyl unit)
	1211	-	1211	-	C-O stretching (phenolic hydroxyl group)
	-	1185 ↑	-	1185 ↑	C=O stretching (acetyl group)
	1106	1106	1106	1106	C-H deformation (syringyl unit)
	1023	1023	1026	1026	C-H deformation and C-O deformation (methoxyl group)
	904	904	908	908	C-H out of plane (aromatic ring)
	847	847	851	851	C-H out of plane (positions 2, 5, and 6 of guaiacyl unit)
Pine wood	3383	3383 ↓	3383	3383 ↓	O-H stretching
	2947	2947	2932	2932	C-H stretching (methoxyl group)
	2825	2825	2825	2825	C-H stretching (methyl and methylene groups)
	-	1734 ↑	-	1734 ↑	C=O unconjugated (carbonyl group)
	1593	1593	1593	1593	Aromatic skeletal vibrations
	1502	1502	1491	1491	Aromatic skeletal vibrations
	1452	1452	1452	1452	C-H deformation (methyl and methylene groups)
	1415	1415	1420	1420	Aromatic skeletal vibrations
	-	1366 ↑	-	1363 ↑	C-H bond (acetyl group)
	1256	-	1256	-	C-O stretching (guaiacyl unit)
	1217	-	1217	-	C-O stretching (phenolic hydroxyl group)
	-	1190 ↑	-	1188 ↑	C=O stretching (acetyl group)
	1143	1143	1136	1136	C-H deformation (guaiacyl unit)
	1036	1036	1026	1026	C-H deformation and C-O deformation (methoxyl group)
	900	900	895	895	C-H out of plane (aromatic ring)
	848	848	856	856	C-H out of plane (positions 2, 5, and 6 of guaiacyl unit)

^1^ Decrease in the intensity of the band. ^2^ Increase in the intensity of the band.

## Data Availability

The original contributions presented in the study are included in the article; further inquiries can be directed to the authors.
